# Speech-Triggered Neurogenic Cough Following Slow Pathway Ablation for Atrioventricular Nodal Reentrant Tachycardia

**DOI:** 10.7759/cureus.110828

**Published:** 2026-06-14

**Authors:** Sebastian Hernandez Mejia, Htat Aung, Rayna Isber, Ahmed S Elcicek, Nidal Isber

**Affiliations:** 1 Internal Medicine, Cardiology, Richmond University Medical Center, New York, USA; 2 Internal Medicine, Richmond University Medical Center, New York, USA; 3 Biology, Barnard College, Columbia University, New York, USA; 4 Electrophysiology, Ondokuz Mayıs University, Samsun, TUR; 5 Electrophysiology, Richmond University Medical Center, New York, USA

**Keywords:** atrioventricular nodal re-entrant tachycardia (avnrt), avnrt ablation, cardiac electrophysiology, chronic cough, neurogenic cough, supraventricular arrhythmias

## Abstract

Persistent cough following catheter ablation for atrioventricular nodal reentrant tachycardia (AVNRT) is not a recognized complication. We report the case of an 81-year-old man who developed a persistent, speech-triggered dry cough beginning the day after successful slow pathway ablation. The cough was reproducibly provoked by phonation, absent during sleep, and persisted despite an unrevealing cardiopulmonary and inflammatory evaluation.

To our knowledge, this phenotype has not been previously described following slow pathway ablation for AVNRT. Unlike post-ablation cough reported in left atrial and pulmonary vein procedures, where pulmonary, esophageal, phrenic, and autonomic mechanisms are well-characterized, slow pathway ablation targets the posteroseptal right atrium, a region not previously implicated in cough-related complications, making this a mechanistically distinct presentation. We propose a post-procedural neurogenic mechanism involving transient sensitization of vagal afferent pathways in the posteroseptal slow pathway region, consistent with a laryngeal hypersensitivity phenotype. Symptoms improved after colchicine, suggesting a limited inflammatory contribution to neural sensitization.

This case expands the spectrum of potential complications after AVNRT ablation and may help guide recognition of atypical post-procedural symptoms while avoiding unnecessary diagnostic evaluation.

## Introduction

Catheter ablation of the slow pathway is a highly effective and widely accepted treatment for atrioventricular nodal reentrant tachycardia (AVNRT), with success rates exceeding 95% and a low rate of major complications [1,2]. Recognized complications most commonly include atrioventricular block and vascular access-related events, whereas persistent cough is not an expected adverse effect.

Cough has been described in association with atrial arrhythmias and in other catheter ablation settings, particularly atrial fibrillation and left atrial procedures, where pulmonary, esophageal, phrenic, and autonomic mechanisms have been proposed [1,2]. The anatomic basis for cough is better understood in those settings. Energy delivery near the pulmonary veins, posterior left atrium, and esophagus can affect structures involved in cough and airway reflexes. Phrenic nerve injury during cryoablation and autonomic effects during ganglionated plexi ablation are also recognized mechanisms. However, this framework does not apply directly to right-sided slow pathway ablation, where lesion delivery targets the posteroseptal right atrium near the coronary sinus ostium, a region distant from the pulmonary veins and esophagus and not previously linked to post-ablation cough. Persistent cough after routine right-sided slow pathway ablation for AVNRT, therefore, appears to be a distinct and poorly described phenomenon.

We report a case of persistent, speech-triggered cough beginning the day after successful slow pathway ablation for AVNRT. To our knowledge, this phenotype has not been previously described in this anatomic and procedural setting. A PubMed search for post-ablation cough following slow pathway ablation for AVNRT identified no comparable reports, supporting the novelty of this presentation. The clinical features and unrevealing cardiopulmonary evaluation support a post-procedural neurogenic mechanism, likely related to transient vagal afferent sensitization with a possible limited inflammatory contribution.

## Case presentation

An 81-year-old man with a 16-year history of symptomatic paroxysmal supraventricular tachycardia was referred for definitive management. His episodes were characterized by the sudden onset of a rapid, regular heartbeat lasting three to four hours and occurring once or twice monthly. Symptoms were occasionally triggered by emotional stress or positional changes. He had previously used Valsalva maneuvers for termination; however, these were discontinued because of associated diverticulitis.

His medical history was notable for diverticulitis and benign prostatic hyperplasia. Medications included tamsulosin and finasteride. He denied tobacco use and reported occasional alcohol consumption. Physical examination was unremarkable, with normal cardiovascular and pulmonary findings. Baseline electrocardiography demonstrated a normal sinus rhythm with normal PR, QRS, and QT intervals and left anterior fascicular block.

An electrophysiology study confirmed typical slow-fast AVNRT. Radiofrequency ablation of the slow pathway was performed via a femoral venous approach under conscious sedation without endotracheal intubation. The procedure was uncomplicated, with successful elimination of inducible tachycardia.

The following day, the patient developed a persistent dry cough that had not been present before ablation. The cough occurred frequently throughout the day and was characteristically triggered by phonation. Even brief speech reliably provoked coughing, to the point that he was often unable to complete a sentence without interruption and had to pause repeatedly while speaking. The cough was absent during sleep. He denied dyspnea, chest pain, fever, sputum production, hemoptysis, recent respiratory infection, medication changes, or gastroesophageal reflux symptoms.

At the two-week follow-up, the cough persisted. Chest radiography demonstrated no acute cardiopulmonary abnormality (Figure 1), and electrocardiography showed normal sinus rhythm with preserved conduction intervals (Figure 2). Physical examination revealed clear lungs and no signs of heart failure or infection. The patient was treated with nonsteroidal anti-inflammatory therapy without improvement in cough frequency or intensity.

**Figure 1:**
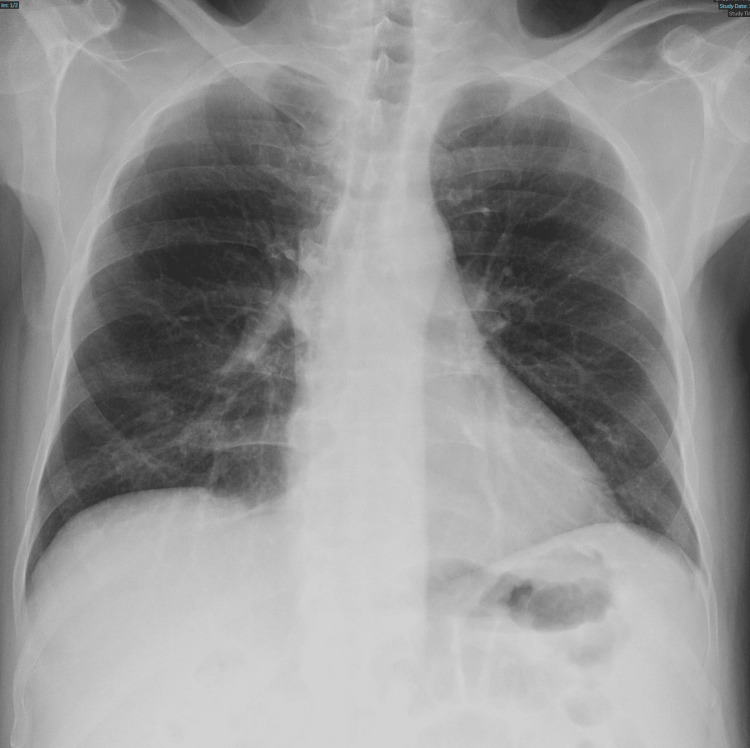
Post-procedural chest radiograph No acute cardiopulmonary abnormality was seen. Lung fields were clear bilaterally without pleural effusion, infiltrate, or cardiomegaly.

**Figure 2:**
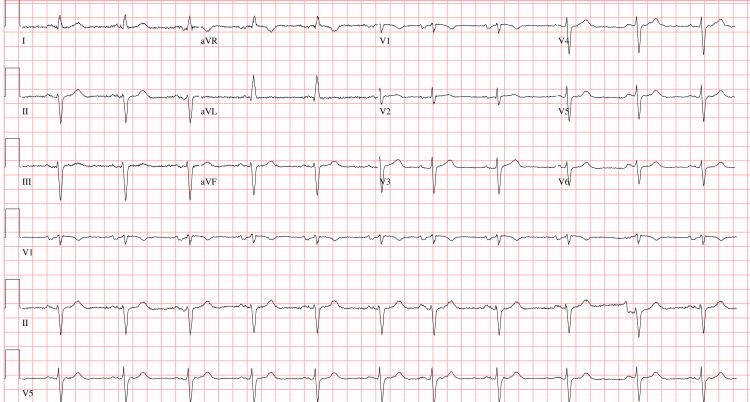
Post-procedure 12-lead electrocardiogram A normal sinus rhythm with a left anterior fascicular block was observed. No evidence of AV nodal conduction abnormalities was noted.

Because symptoms persisted, the patient was referred to pulmonology and further evaluation was pursued. Pulmonary function testing and allergy evaluation were unremarkable. Laboratory testing, including complete blood count, C-reactive protein, and erythrocyte sedimentation rate, was unremarkable. Noncontrast chest computed tomography demonstrated no acute thoracic abnormality or structural explanation for cough. Post-procedural transthoracic echocardiography was also unremarkable, without pericardial effusion or other structural complication. Empirical trials of a proton pump inhibitor and cetirizine were initiated without improvement. Medication review did not identify an obvious pharmacologic cause of cough.

Given the close temporal relationship to ablation, the absence of intubation, and the lack of evidence for pulmonary, infectious, pericardial, structural cardiac, or medication-related causes, a post-procedural neurogenic cough with possible limited inflammatory contribution was suspected. At the 6-week follow-up, colchicine 0.6 mg daily was initiated empirically. Within three days, he reported significant improvement, with reduced cough frequency and improved ability to speak without triggering symptoms. At subsequent follow-up, he remained free of palpitations and reported continued improvement in cough.

## Discussion

This case highlights an exceptionally rare but clinically relevant complication of slow pathway ablation: a persistent, speech-triggered cough most consistent with a post-procedural neurogenic mechanism. Although slow pathway ablation is highly effective and generally low risk, with success rates exceeding 95%, persistent cough is not a recognized expected complication [1,2]. To our knowledge, no prior PubMed-indexed report has described this phenotype after slow pathway ablation for AVNRT. The closest report we found was post-cardiac injury syndrome after right septal ablation, which represents a different inflammatory presentation characterized by fever, pericardial effusion, and elevated inflammatory markers rather than isolated phonation-triggered cough [3].

Post-ablation cough has been reported more often after left atrial and pulmonary vein isolation procedures, where lesion delivery occurs near the posterior left atrium, pulmonary vein ostia, and adjacent esophagus, all of which lie close to structures involved in pulmonary, phrenic, and vagal cough reflexes. In contrast, similar reports after right-sided slow pathway ablation are notably scarce, suggesting that anatomy may contribute to the distinctiveness of this case. Slow pathway ablation targets the posteroseptal right atrium near the coronary sinus ostium, where the inferior right atrial ganglionated plexus, a recognized autonomic structure containing vagal afferent fibers, is located [4,5]. Radiofrequency delivery in this region may have transiently sensitized these pathways and lowered the threshold for cough reflex activation. Parasympathetic responses during radiofrequency delivery at right atrial sites near the slow pathway region have also been described, further supporting the biologic plausibility of this mechanism [4,5].

The cough pattern fits this interpretation. Reproducible triggering by phonation, severity enough to interrupt speech, and absence during sleep are features consistent with cough hypersensitivity syndrome, which the European Respiratory Society describes as troublesome coughing triggered by low-level stimuli and reflecting abnormal vagal sensory function rather than primary structural airway disease [6]. Alternative explanations were excluded. The procedure was performed without intubation, making airway trauma less likely, and chest radiography, computed tomography, echocardiography, and inflammatory markers were unrevealing, arguing against pulmonary, infectious, pericardial, structural cardiac, or medication-related causes.

Although overt post-cardiac injury syndrome was unlikely, given the absence of chest pain, pericardial effusion, and elevated inflammatory markers, a limited inflammatory contribution to neural sensitization may still have been present. The lack of response to nonsteroidal anti-inflammatory drug (NSAID) therapy is relevant because NSAIDs inhibit cyclooxygenase-mediated prostaglandin synthesis but do not affect the NLRP3 inflammasome-IL-1β pathway, which has been linked to perineural inflammation and lower afferent sensory thresholds [7]. Colchicine, in contrast, interferes with microtubule-dependent inflammasome assembly and neutrophil migration, targeting an upstream inflammatory pathway that NSAIDs do not address [7].

The improvement after colchicine should not be taken as evidence of pericarditis, but it raises the possibility that limited perineural inflammation contributed to vagal afferent sensitization. Limitations should be acknowledged. First, spontaneous resolution at six weeks cannot be excluded, and no rechallenge was performed. Second, the use of colchicine in this setting remains hypothesis-generating rather than evidence-based. Third, objective cough quantification and formal laryngoscopic assessment were not obtained. Finally, direct autonomic or neurophysiologic confirmation of vagal afferent sensitization was not available, and the proposed mechanism remains inferential.

Recognition of this presentation is important because it expands the spectrum of symptoms that may follow AVNRT ablation and may help clinicians avoid unnecessary testing when a similar cough develops shortly after slow pathway ablation, especially when it is speech-triggered and occurs without other signs of pulmonary or pericardial disease. More broadly, this case highlights a gap in the post-ablation literature: persistent cough has been described mainly in left atrial ablation settings, whereas its occurrence after right-sided slow pathway ablation remains poorly defined. Further study is needed to better understand the incidence, mechanisms, and management of this underrecognized post-ablation phenomenon.

## Conclusions

Persistent, speech-triggered cough after slow pathway ablation for AVNRT appears to be an exceptionally rare and likely underrecognized complication. This case supports a post-procedural neurogenic mechanism, likely related to transient vagal-autonomic sensitization in the posteroseptal slow pathway region, with a possible limited inflammatory contribution. Recognizing this phenotype may help clinicians avoid unnecessary testing and broaden awareness of post-ablation cough after right-sided slow pathway ablation.
